# Emerging Molecular PET Imaging for Cell Death in Neurodegenerative Diseases

**DOI:** 10.1021/acs.jmedchem.5c01512

**Published:** 2025-10-28

**Authors:** Jing-Jing Zhang, Jing Yang, Jimmy S. Patel, Rui Lv, Zhijie Zhang, Xin Ji, Lijun Tang, Zhen Chen, Steven H. Liang

**Affiliations:** 1 State Key Laboratory for Development and Utilization of Forest Food Resources, Jiangsu Co-Innovation Center of Efficient Processing and Utilization of Forest Resources, 74584 Nanjing Forestry University, Nanjing, Jiangsu 210037, China; 2 School of Engineering, China Pharmaceutical University Nanjing 210009, China; 3 Department of Radiology and Imaging Sciences, 1371 Emory University, 1364 Clifton Rd, Atlanta, Georgia 30322, United States; 4 Department of Nuclear Medicine, The First Affiliated Hospital with Nanjing Medical University, Nanjing, Jiangsu 210029, China

## Abstract

Neuronal cell death is closely associated with various neurodegenerative diseases. The ability to visualize neuronal cell death is critical for facilitating early diagnosis, monitoring disease progression, evaluating therapeutic efficacy, and developing new treatments for these conditions. Positron emission tomography (PET), as a noninvasive imaging tool, enables real-time visualization of biochemical processes *in vivo* with quantitative information, offering valuable insights into cell death mechanisms in neurodegeneration. This perspective highlights recent advances in PET probes developed to visualize cell death in neurodegenerative diseases, as well as emerging probes targeting mechanisms associated with various forms of neuronal cell death. We anticipate that this perspective will inspire the development of PET imaging probes for the clinical diagnosis and treatment of neurodegenerative diseases.

## Graphical abstract

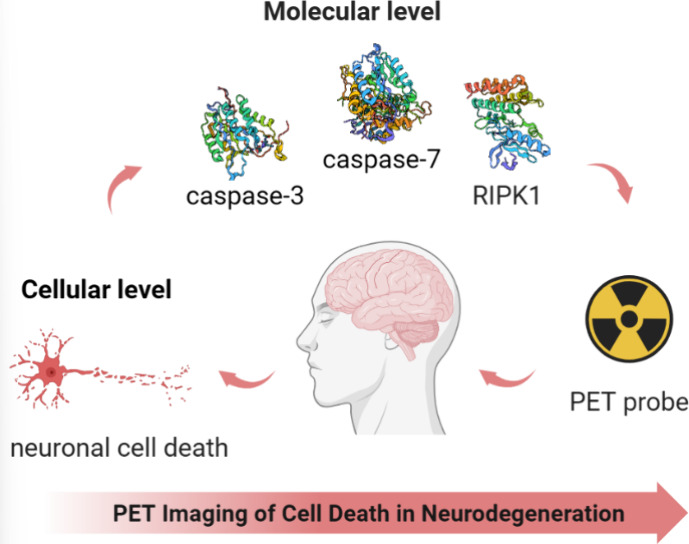

## 1 Introduction

Neurodegenerative diseases are characterized by the progressive deterioration of neuronal function, driven by synaptic and axonal degeneration, ultimately leading to neuronal cell death.^1^ Neurodegenerative diseases encompass well-known conditions such as Alzheimer’s disease (AD), frontotemporal lobar degeneration (FTLD), and Lewy body dementia (LBD), as well as Parkinson’s diseases (PD), amyotrophic lateral sclerosis (ALS), Huntington’s disease (HD), and Creutzfeldt–Jakob disease (CJD). These conditions affect millions of individuals worldwide, leading to progressive cognitive and motor impairments, reduced quality of life, and significant socioeconomic burden.^1^ Currently, no effective treatments are available to slow the progression in a majority of these diseases.

Neuronal cell death is a crucial factor in the pathogenesis of neurodegenerative diseases, with aberrant neuronal cell death implicated in a variety of these disorders.^1–5^ Traditionally, apoptosis and necrosis have been considered the primary pathways of neuronal death.^6^ However, emerging evidence indicates that other pathways, such as ferroptosis, autophagy, paraptosis, pyroptosis, and parthanatos are also involved.^2^ Monitoring neuronal cell death in patients with neurodegenerative diseases and identifying its specific mechanism can guide a physician to prescribe the correct inhibitor to delay or prevent cell death to save the neuron and timely adjustment of treatment plans, thereafter delaying or treating disease progression and reducing healthcare costs.^7–18^

Positron emission tomography (PET) serves as a powerful, sensitive and noninvasive tool for visualizing cellular processes in vivo.^19–25^ Distinct cell death pathways exhibit unique morphological features and signaling mechanisms,^2,3,26^ offering opportunities to develop PET probes that target specific biomarkers or biochemical processes associated with neuronal cell death.^27,28^ Examples include caspases, receptor-interacting protein kinase 1 (RIPK1), poly­(ADP-ribose) polymerase (PARP), phosphatidylserine (PS), and phosphatidylethanolamine (PE). Several PET probes for imaging cell death have advanced to clinical application, such as ^18^F-labeled 5-fluoropentyl-2-methyl-malonic acid ([^18^F]­ML-10) for apoptotic membrane imprint.^29^ However, most of these tracers have focused on oncologic applications, with limited assessment, comparatively, for neurodegenerative diseases.^30^ One of the primary obstacles is the blood–brain barrier (BBB), which restricts the delivery of many imaging agents to the central nervous system (CNS). For example, in a phase II clinical trial, [^18^F]­ML-10 successfully visualized apoptotic cell death in infarcted brain regions of patients with acute ischemic stroke (day 3 postonset),^31^ where the BBB is typically disrupted. However, its limited ability to cross an intact BBB restricted its uptake to areas showing contrast enhancement on MRI.^32^

This perspective summarizes PET probes reported in the past decade with potential for monitoring neuronal cell death. In addition to PET tracers specifically designed for cell death imaging in neurodegenerative disease, we also include representative tracers targeting molecules involved in cell death in tumors but with BBB-penetrating capability. Given the crosstalk among different cell death pathways,^2,26^ the PET tracers are introduced based on the biomarkers of cell death rather than the specific mechanisms of cell death. Furthermore, we discuss the radiosynthesis of radiotracers, their pharmacology, in vivo validation, and the challenges encountered in developing these PET probes.

## 2 Cell Death Pathways in Neurodegenerative Diseases

Different modes of cell death, which include extrinsic and intrinsic apoptosis, necroptosis, parthanatos, ferroptosis, pyroptosis, oncosis, lysosomal, autophagic, phagocytic, and mitoPore, have been linked to neurodegenerative diseases.^2^ Although each cell death pathway is associated with distinct physiological changes, considerable overlap in their features often makes differentiating between them challenging. Apoptosis is classically characterized by changes in the expression of Bcl-2 family proteins, activation of caspases, DNA fragmentation, and externalization of PS. However, many of these molecular hallmarks are shared across other cell death pathways. For example, Bcl-2 family proteins also play a role in regulating autophagy, and caspase activation is observed in both pyroptosis and certain forms of necrosis.^2^ Similarly, [^18^F]­FDG is used to detect increased cellular metabolism of glucose. In metabolically active tissues such as the brain, it is inherently challenging to distinguish pathological processes using imaging, as high baseline metabolic activity can mask disease-specific signals. Similarly, PET tracers that target general aspects of cell physiology may lack the specificity needed to discriminate between distinct cell death pathways. This ambiguity can complicate both diagnosis and the selection of targeted therapeutic strategies, such as choosing the appropriate cell death inhibitor. To address this challenge, a promising alternative is the development of radiotracers that target key molecular markers unique to specific cell death mechanisms. Several such neuronal cell death pathways relevant to neurodegenerative diseases, and their corresponding molecular targets, have been comprehensively discussed.^1–3^ Herein, the role of each marker in cell death will be explained in brief with a subsequent discussion on their corresponding PET targeting probes.

## 3 PET Tracers for Imaging Cell Death in the Brain

### 3.1 PET Tracers Targeting Caspase

Caspase is a family of cysteine aspartate-directed proteases, which plays an important role in several cell death mechanisms such as extrinsic apoptosis, intrinsic apoptosis and pyroptosis. The extrinsic apoptotic pathway is triggered by the activation of death receptors, followed by the downstream activation of the key initiator caspase, caspase-8. On the other hand, the intrinsic pathway is normally promoted by the cellular stress which leads to downstream release of cytochrome, and subsequent activation of initiator caspase-9. Both of these mechanisms eventually merge in the activation of executioner caspases such as caspase-3 and caspase-7.^33,34^ As a result, caspase-3 and caspase-7, which share significant homology, have been hypothesized to be potential targets by PET radiotracers to image apoptosis.^34–36^ Caspase specific radiotracers have been designed to bind to activated caspases, or to be caspases-cleavable as a reporter-like mechanism. Although a variety of PET probes for caspase-3 have been reported, few have been designed to image cell death in neurodegenerative disease, due to presence of the BBB.^34^

The isatin sulfonamide family is one of the most extensively studied groups of radiotracers targeting caspases.^34^ These tracers exhibit high affinity for caspase-3/7, attributed to the formation of a reversible covalent bond through the nucleophilic attack of the active-site cysteine residue (Cys163) in caspases at the 3-carbonyl group of the isatin ring. Sobrio and colleagues reported a series of isatin compounds [^18^F]**1**-**3** (Figure 1) with improved affinity for caspase-3/7 and enhanced *in vivo* stability.^35^ These tracers can be readily prepared on an automated GE TracerLab FX-FN module via nucleophilic substitution, where fluorine-18 is introduced by displacement of a sulfonate leaving group in the isatin precursor. High radiochemical purity (>98%) and high specific radioactivity (220–270 GBq/μmol) were achieved. The IC_50_ values for caspase-3 were 8.4, 8.1, and 13.7 nM, and for caspase-7, 4.6, 5.4, and 9.6 nM, respectively, for these isatin PET tracers. *In vivo* imaging of apoptosis using these radiotracers was evaluated in a rat stroke model by temporary middle cerebral artery occlusion (MCAO) without craniectomy. In this model, the expression of activated caspase-3 reached the maximal value at 24 h postischemia. As a result, *in vivo* PET imaging was investigated 24 h after the ischemic stroke was induced. Initial PET imaging at standard resolution showed generally weak uptake in both ischemic and contralateral (unaffected) brain regions. To further investigate, microPET (μPET) imaging was performed, which offers higher spatial resolution and sensitivity for small-animal studies. Notably, μPET revealed significant differences in uptake between the ischemic and contralateral regions between 20 and 45 min for [^18^F]**2** and after 45 min for [^18^F]**1**. Uptake ratio for the regions of interest (ROI) increased up to 1.52 at 90 min for [^18^F]**1** and reached to 1.26 at 62 min for [^18^F]**2**. In contrast, no clear differences were observed for [^18^F]**3** or the reference tracer [^18^F]­ML-10, a well-established apoptosis imaging agent targeting apoptotic cell membrane changes.

**Figure 1:**
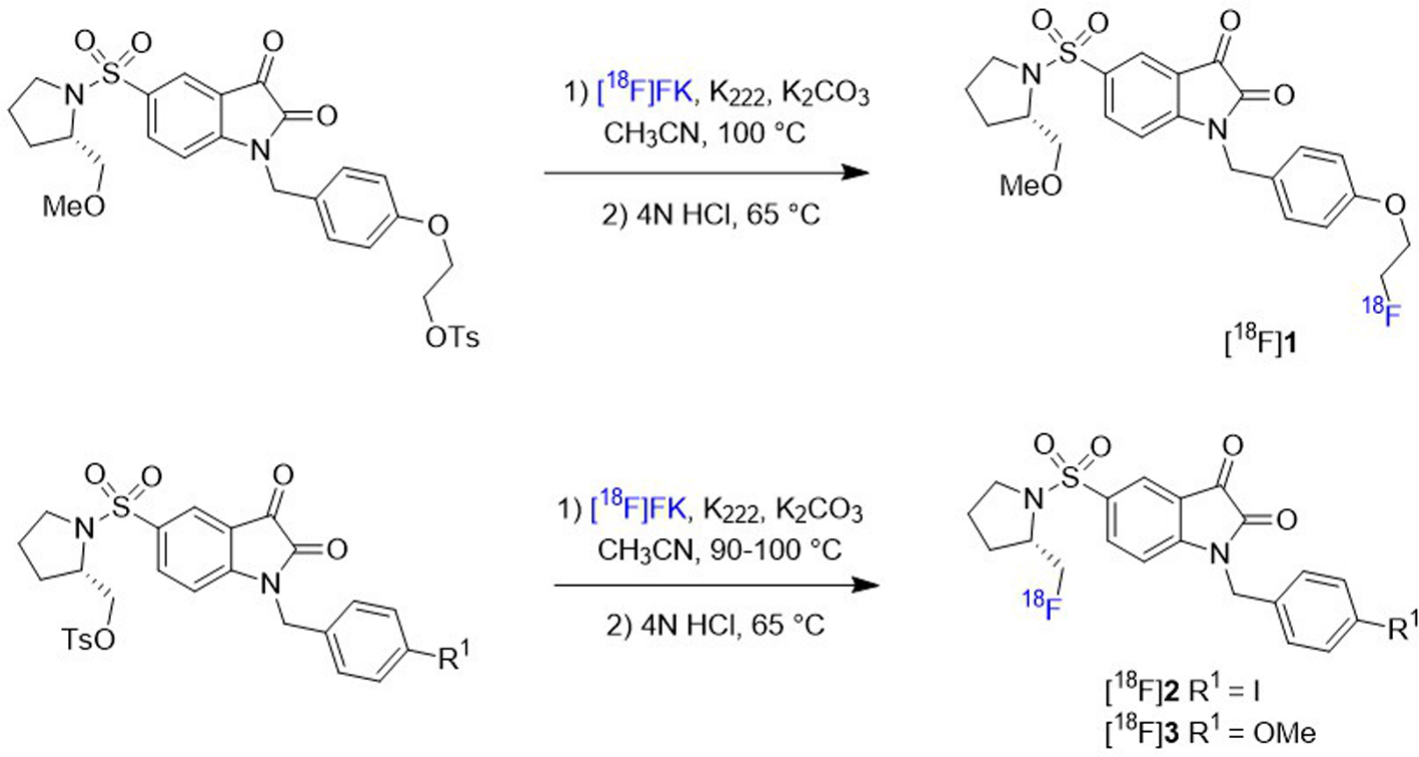
Radiosynthesis of [^18^F]**1**-**3**.

Bartha reported a dual PET/fluorescent tracer [^68^Ga]­Ga-TC3-OGDOTA, which can detect active caspase-3 both *in vitro* and *in vivo*.^33^ As depicted in Figure 2A, this dual-modality tracer was synthesized in two steps. First, a caspase-cleavable peptide was labeled with the fluorescent dye Oregon Green via nucleophilic substitution at its cysteine side chain. In the second step, the peptide was radiolabeled by coordinating its DOTA moiety with [^68^Ga]­GaCl_3_ to produce the final PET tracer. Rodent models of neurological models, including stroke (MCAO rat) and AD (5xFAD mouse), were used to study the *in vivo* PET imaging. The 5xFAD model displayed obvious neurodegeneration and caspase-3 activity in the mice by the age of 8–9 month. Therefore, mice older than 8 months were applied in this model for PET imaging study. [^68^Ga]­Ga-TC3-OGDOTA demonstrated brain accumulation in both rodent models. However, the resolution of [^68^Ga]­Ga-TC3-OGDOTA distribution in mouse tissues is too low to isolate uptake from specific brain structures. Instead, a simplified assignment of ROIs was employed. For instance, in the 5xFAD model, PET kinetic analysis suggested preferred tracer accumulation in the forebrain, which was further supported by a positive Patlak *K*_i_ constant using the hindbrain as the reference region (Figure 2B). The uptake pattern overlapped with Thioflavin S staining, indicating diffuse localization around amyloid plaques. Moreover, [^68^Ga]­Ga-TC3-OGDOTA successfully identified apoptotic neurons both in vitro and in vivo. Optical clearing revealed colocalization of [^68^Ga]­Ga-TC3-OGDOTA with active caspase-3 in brain cells (marked with white arrows in Figure 2C).

**Figure 2:**
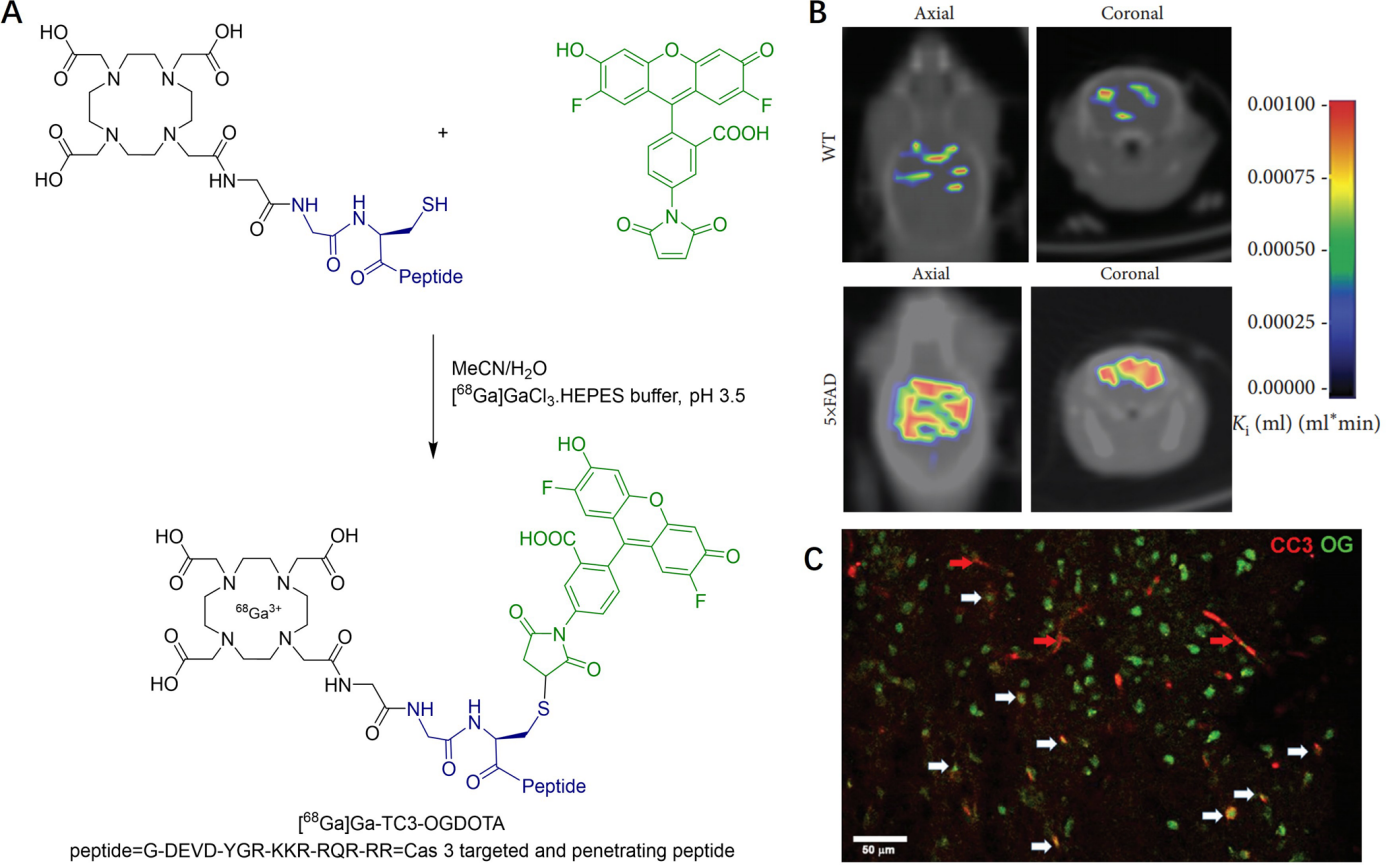
(A) Preparation of radiolabeled probe [^68^Ga]­Ga-TC3-OGDOTA, (B) Representative coronal and axial sections of K_i_ mapping in a wild-type (WT) and a 5xFAD mouse brains. *K*_i_ map was aligned with a CT mouse image manually, and (C) fluorescence images for cleaved caspase-3 (red) and [^68^Ga]­Ga-TC3-OGDOTA (green) in brains extracted from [^68^Ga]­Ga-TC3-OGDOTA-treaed MCAO mice. Reprinted with permission under CC BY 4.0 from ref 33, Copyright © 2019 John Wiley & Sons Ltd.).

Although the development of caspase-targeting tracers with enhanced BBB permeability has significantly expanded their potential beyond oncology, their translation to neurodegenerative disease imaging remains in early stages, with challenges including limited caspase activation levels, transient expression patterns, and off-target effects. Continued optimization in tracer design and validation in relevant neurodegenerative models will be essential to fully realize its diagnostic value in CNS disorders.

### 3.2 PET Tracers Targeting RIPK1

RIPK1, a death-domain containing serine/threonine kinase, plays an important role in regulating cell death. Activation of RIPK1 via tumor necrosis factor receptor 1 (TNFR1) can trigger either extrinsic apoptosis or necroptosis. RIPK1-dependent cell death has been closely associated with neurodegenerative diseases such as ALS, FTLD, and AD.^37–40^ Structurally, RIPK1 features a distinctive hydrophobic pocket within its kinase-regulating allosteric domain, making it an attractive target for pharmacological inhibition.^37^ The development of PET radiotracers in this area leverages the diverse pool of existing RIPK1 inhibitors. Several other efforts have encountered challenges related to poor brain permeability or nonspecific binding. For example, although [^11^C]**PK68** (Figure 3) showed promise as a RIPK1-targeting PET probe, it struggled to effectively penetrate the brain.^41^ Another tracer, [^11^C]**GG502** (Figure 3), demonstrated specific binding to RIPK1 and good brain permeability but suffered from rapid in vivo metabolism, leading to nonspecific binding signals.^42^

**Figure 3:**
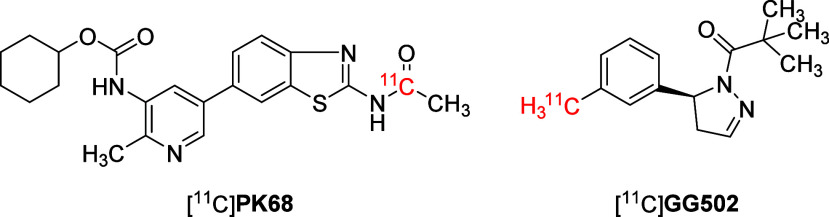
Structures of [^11^C]**PK68** and [^11^C]**GG502**.

Zhang et al. developed the RIPK1-targeting PET tracer [^18^F]**CNY-07** (*K*_d_ = 68 nM) (Figure 4A), through copper-mediated [^18^F]­fluorination of the aryl boronate ester precursor, based on a small-molecule RIPK1 inhibitor 5-((1H-indol-3-yl)­methyl)-3-methyl-2-thioxoimidazolidin-4-one (**Nec-1**) and its analog **Nec-1s** (Figure 4B).^43^ [^18^F]**CNY-07** was obtained in good radiochemical yield (RCY) (23–28%, nondecay-corrected to trapped [^18^F]­fluoride) and high radiochemical purity (>95%) with a specific activity of 1.766 ± 0.2 Ci/μmol. In vivo PET imaging in rodents demonstrated that [^18^F]**CNY-07** was able to effectively penetrate the BBB (Figure 4C), reaching a maximum percent injected dose per unit volume (%ID/cc) of 3 in the brain of mice at 10 min postinjection, with acceptable in vivo metabolic stability. Although [^18^F]**CNY-07** exhibited relatively high affinity for RIPK1 with *K*_d_ of 68 nM, the brain uptake of [^18^F]**CNY-07** appeared to reflect somewhat limited in vivo binding specificity rather than RIPK1-specific binding, as indicated by only 30% reduction in brain uptake in self-blocking experiments.^43^ Additionally, [^18^F]**CNY-07** exhibited relatively slow clearance, with brain radioactivity remaining at ∼ 3%ID/cc even at 60 min postinjection, which may hamper translational potential.

**Figure 4:**
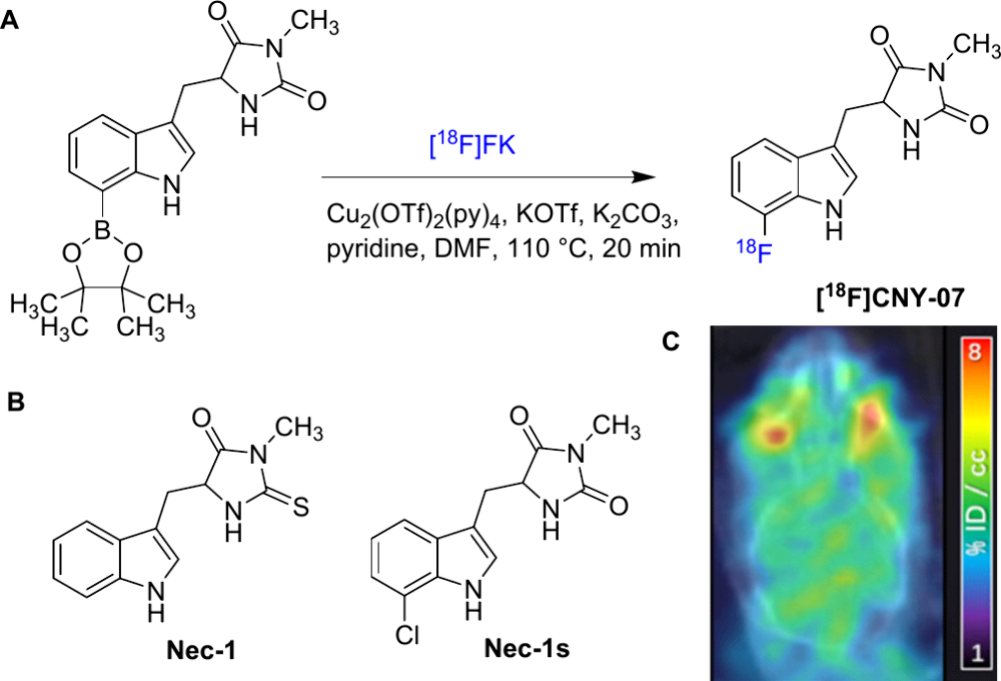
(A) Radiosynthesis of [^18^F]**CNY-07**, (B) the structures of **Nec-1** and **Nec-1s**, and (C) PET imaging of [^18^F]**CNY-07** in mouse brain (Reprinted with permission from ref 43, Copyright © 2021 American Chemical Society).

To address the clearance limitations of [^11^C]**CNY-07**, Bai and co-workers developed a second-generation molecule, [^11^C]**CNY-10** (also known as [^11^C]**Nec-1s**).^44^ [^11^C]**CNY-10** was synthesized via a single-step *N*-methylation reaction using [^11^C]­methyl iodide (Figure 5A). The tracer was obtained with high radiochemical purity (>95%) and excellent molar activity (305 GBq μmol^–1^). Compared to [^18^F]**CNY-07**, [^11^C]**CNY-10** demonstrated increased brain uptake (SU*V*_max_: 0.8 vs 0.6) and improved clearance kinetic profile in wild-type mice, with brain radioactivity decreasing from 0.8 SUV at 10 min to 0.3 SUV at 60 min postinjection, indicating faster washout. High brain uptake and heterogeneous distribution of [^11^C]**CNY-10** was also observed in nonhuman primates (NHP). Additionally, 8-month-old 5xFAD mice were used to study the performance of [^11^C]**CNY-10** in AD mice. In 5xFAD mice model, [^11^C]**CNY-10** showed promising potential to visualize neurodegenerative disease-related changes, as evidenced by higher brain uptake across all ROIs in the 5xFAD mice compared to wild-type controls (Figure 5B). Importantly, significant increase of RIPK expression was also observed across brain areas investigated in AD mice compared to WT mice. Taken together, [^11^C]**CNY-10** emerges as a valuable RIPK1-targeting PET radioligand, warranting further translational investigation.

**Figure 5:**
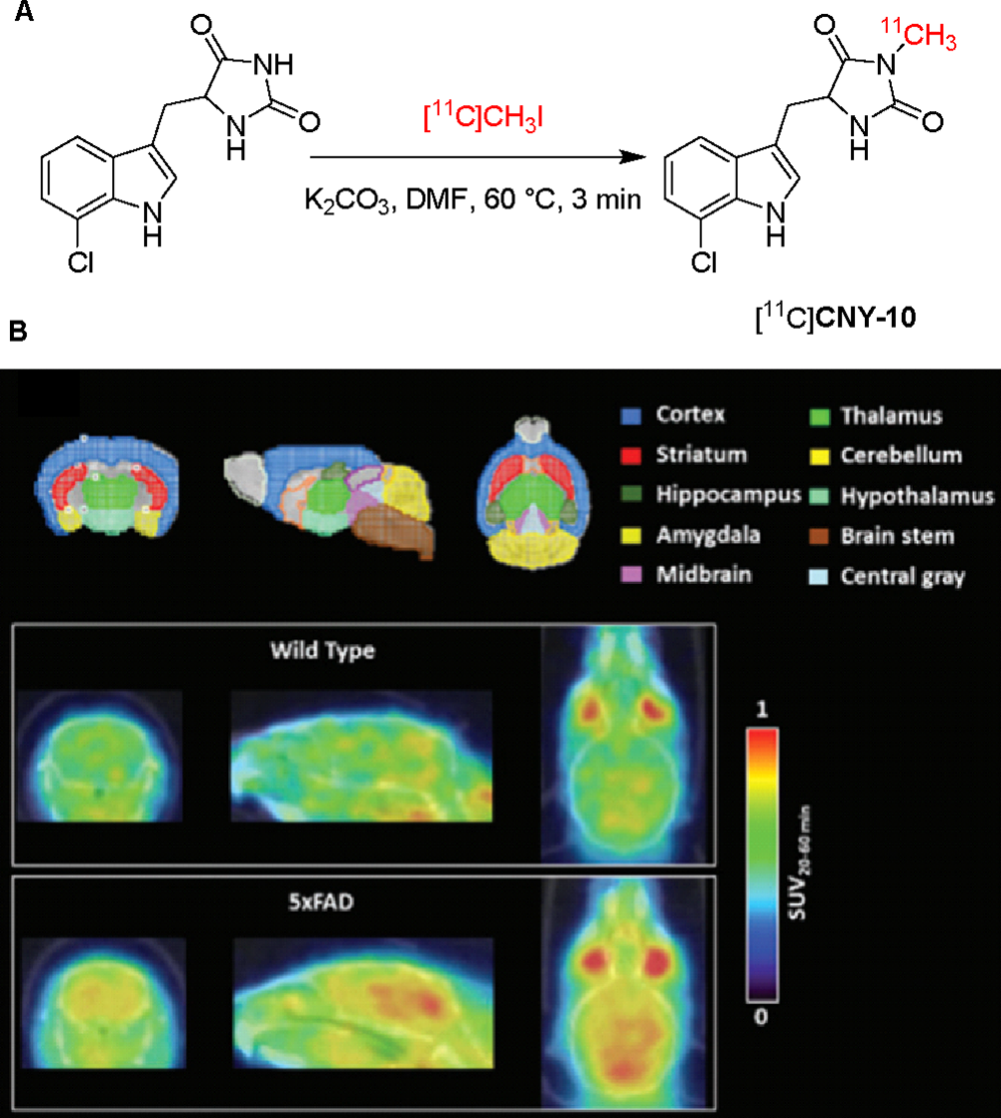
(A) Radiosynthesis of [^11^C]**CNY-10** and (B) Brain distribution of [^11^C]**CNY-10** in 5xFAD mice and WT mice (Reprinted with permission from ref 44, Copyright © 2024 Wiley-VCH GmbH).

Compared to the first-generation of RIPK1 inhibitors such as Nec-1 and its variants, 7-oxo-2,4,5,7-tetrahydro-6H-pyrazolo­[3,4-*c*]­pyridine (**TZ7774**) has emerged as a more potent RIPK1 inhibitor with a *K*_i_ of 0.91 nM.^45^ Based on this scaffold, Tu’s group developed a novel RIPK1-specific PET radiotracer, [^11^C]**TZ7774**, by radiolabeling **TZ7790**, a demethylated variant of **TZ7774**, with [^11^C]­methyl iodide via *N*-[^11^C]­methylation under basic condition (Figure 6A).^45^ [^11^C]**TZ7774** was produced with high RCY (30–40%, 1.85 GBq, decay corrected to end of bombardment (EOB)), good chemical purity (>90%), excellent radiochemical purity (>99%), and high molar activity (>207 GBq/μmol). The tracer demonstrated good BBB penetration, achieving a brain uptake of 0.53%ID/g in Sprague–Dawley rats at 5 min postinjection, and showing promising initial brain uptake in NHPs with a SUV of ∼ 3.7 at 6–10 min postinjection, suggesting that [^11^C]**TZ7774** holds potential for RIPK1-targeted PET imaging (Figure 6B).

**Figure 6:**
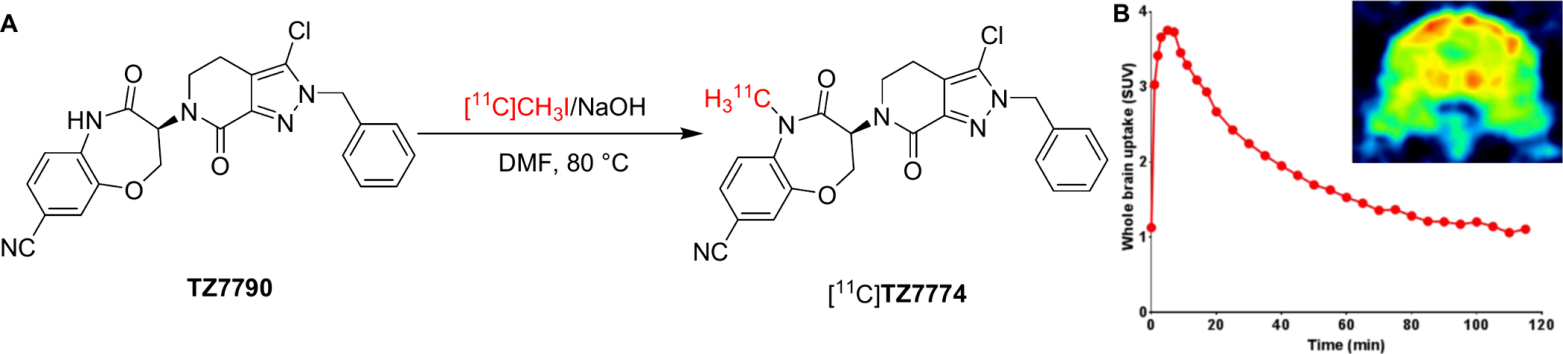
(A) Radiosynthesis of [^11^C]**TZ7774** and (B) PET imaging of [^11^C]**TZ7774** in macaque brain (Reprinted with permission from ref 45, Copyright © 2022 Elsevier Inc.).

To further enhance the accuracy and precision of RIPK1-specific PET radiotracers, Bai and co-workers developed two analogs of [^11^C]**TZ7774**: 7-Oxo-2,4,5,7-tetrahydro-6H-pyrazolo­[3,4-*c*]­pyridine-based tracers [^11^C]**PB218** and [^11^C]**PB220**.^46^ Both tracers demonstrated potent antinecroptosis activity, with EC_50_ values of 0.62 nM for [^11^C]**PB218** and 0.66 nM for [^11^C]**PB220** in a TZ-induced necroptosis model using human U937 cells. The radioligands were synthesized via radiolabeling of their respective precursors with [^11^C]­CH_3_I, achieving good RCYs (34%–42%, n = 6, nondecay corrected to trapped [^11^C]­CH_3_I), high molar activity (293–314 GBq/μmol), and excellent radiochemical purity (>98%) (Figure 7A). The 60 min dynamic PET imaging in rodents demonstrated good brain uptake with a SUV of 1.0 and 0.9 for [^11^C]**PB218** and [^11^C]**PB220**, respectively. Notably, [^11^C]**PB218** showed superior binding specificity in self-blocking experiments. Its promising translational potential was further supported by high brain penetration (Figure 7B) and favorable kinetic profiles observed in NHPs, underscoring its suitability for RIPK1 imaging in the brain.

**Figure 7:**
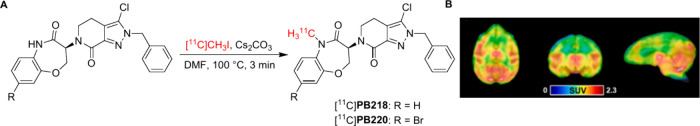
(A) Radiosynthesis of [^11^C]**PB218** and [^11^C]**PB220**, (B) Brain uptake of [^11^C]**PB218** in NHP (Reprinted with permission from ref 46, Copyright © 2024 Elsevier Inc.).

Recently, Fu and co-workers reported a series of potent dihydropyrazole-cored RIPK1 PET ligands.^47,48^ Among these ligands, [^18^F]**WL1** (Figure 8A) displayed the greatest potential for specific binding to RIPK1 in the brain. [^18^F]**WL1** was prepared via a Cu­(II)-mediated aromatic nucleophilic substitution reaction of the corresponding pinacolboronic ester precursor with good isolated RCY (28.6%, decay-corrected), high radiochemical purity (>99%) and good molar activity (2.1 GBq/μmol) (Figure 8A). [^18^F]**WL1** not only displayed specific binding activity to RIPK1 in mouse brain sections *in vitro*, but also showed a high initial brain uptake (4.89% ID/g at 2 min) and fast washout (0.21% ID/g at 60 min). Moreover, in the dynamic PET imaging study, the radioactivity uptake of [^18^F]**WL1** in the brain decreased by 21.7% upon self-blockade and reduced by 25.9% upon blockade with the structurally diverse blocking agent GSK’547 (Figure 8B), suggesting the potent specific binding of [^18^F]**WL1** in the rat brain. Furthermore, a TNFα-induced systemic inflammatory response syndrome model was used to investigate the expression change of RIPK1. [^18^F]**WL1** was administrated 3.5 h after rats were intravenously injected TNFα solution. In this model, the radioactivity uptake of [^18^F]**WL1** in rat brains increased by 9.8% due to self-blocking, which further confirmed the potential of [^18^F]**WL1** for RIPKI imaging in the brain.

**Figure 8:**
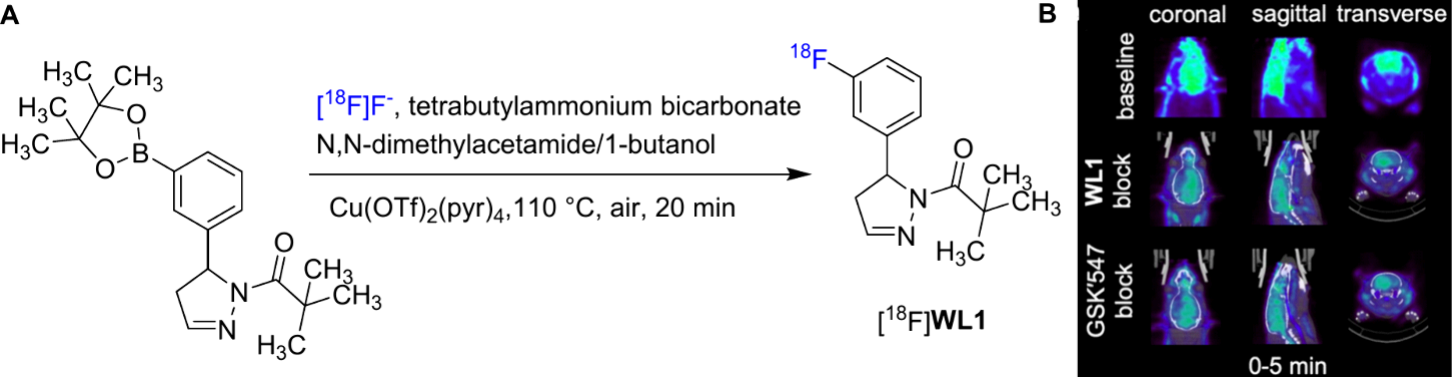
(A) Radiosynthesis of [^18^F]**WL1**, (B) PET images of [^18^F]**WL1** in the brain sections of rats under baseline and blocking conditions (Reprinted with permission from ref 47, Copyright © 2024, American Chemical Society).

### 3.3 PET Tracers Targeting Phosphatidylserine (PS)

Phosphatidylserine (PS) is a negatively charged phospholipid that is predominantly confined to the inner leaflet of the plasma membrane under normal physiological conditions, maintained by ATP-dependent flippase enzymes.^49,50^ PS externalization is a hallmark of apoptosis and has been extensively studied in peripheral diseases, including oncology and cardiovascular injury.^51^ However, in the context of neurodegeneration, PS exposure is far more complex. Neuronal apoptosis is often incomplete or dysregulated, with features overlapping other cell death modalities such as necroptosis and ferroptosis. Despite this, PS remains a potential imaging target due to its early appearance in apoptotic signaling and accessibility on the outer membrane. Importantly, PS exposure has been observed in post-mortem samples from AD and ALS patients, particularly in regions with high microglial activation, suggesting that PS-targeting probes could, in theory, capture early neuronal stress responses prior to complete cell demise.^52,53^

Annexin V, a calcium-dependent phospholipid-binding protein, exhibits high affinity for PS with a *K*_d_ of approximately 10 nM.^53^ Leveraging this strong and specific interaction, fluorophore- and radiolabeled Annexin V and its derivatives have been extensively used to assess apoptosis in both preclinical and clinical settings.^54^ Radiolabeled Annexin V peptides represent the first generation of tracers developed for *in vivo* imaging of cell death, capable of detecting both apoptotic and necrotic processes.^55,56^ Despite their utility, the application of Annexin V-based tracers in neurodegenerative disease has been significantly limited by their large molecular size (∼36 kDa), which impedes their ability to cross BBB, thus preventing effective imaging of neuronal cell death in the CNS.

Smith and colleagues developed a small-molecule, fluorescent Zn^2+^-2,2’-dipicolylamine (Zn^2+^-DPA) complex as a biomimetic alternative to Annexin V, which demonstrated enhanced potential for *in vitro* apoptosis imaging by offering faster binding kinetics, smaller molecular size for improved tissue penetration, and less dependence on calcium concentration.^57^ Unlike Annexin V, which requires micromolar levels of extracellular calcium to maintain binding, Zn^2+^-DPA retained high affinity for PS-rich apoptotic cell membranes even under variable calcium conditions, providing more robust and versatile imaging. Building on this, Wang’s group advanced the complex for PET imaging by developing [^18^F]**FP-DPAZn2**.^58^ As shown in Figure 9A, [^18^F]­NFP, a widely used prosthetic group for radiolabeling peptides, was first synthesized using a modified PET-MF-2 V-IT-I procedure, achieving a decay-corrected RCY of 35 ± 5% (n = 10) from [^18^F]­fluoride.^59^ [^18^F]**FP-DPAZn2** was then produced by coupling the precursor **DPA2** with [^18^F]**NFP**, resulting in a decay-corrected RCY of 90 ± 5% (n = 10) from [^18^F]**NFP** within 30 min, and an overall RCY of 30 ± 10% (n = 10) from [^18^F]­fluoride over 110 min. The final product exhibited high radiochemical purity (>95%) and molar activity (4.0 GBq/μmol). Initially designed for imaging cardiomyocyte apoptosis, [^18^F]**FP-DPAZn2** demonstrated moderate brain uptake (1.12 ± 0.18% ID/g at 30 min postinjection) in normal rats. Afterward, 7-month-old double transgenic AD mice from B6C3-Tg (APPswe, PSEN 1dE9) 85 Dbo/J mice were used for AD model imaging of [^18^F]**FP-DPAZn2**.^60^ Increased accumulation of [^18^F]**FP-DPAZn2** was clearly observed in the brain of AD model mice at 18 min postinjection compared to normal mice with an uptake ratio of 1.35, and the uptake ratio increased up to 1.88 at 60 min (Figure 9B). However, the moderate brain uptake of [^18^F]**FP-DPAZn2** underscores the need for rational structural optimization, such as increasing lipophilicity or introducing transporter ligands, to enhance its CNS imaging applications.

**Figure 9:**
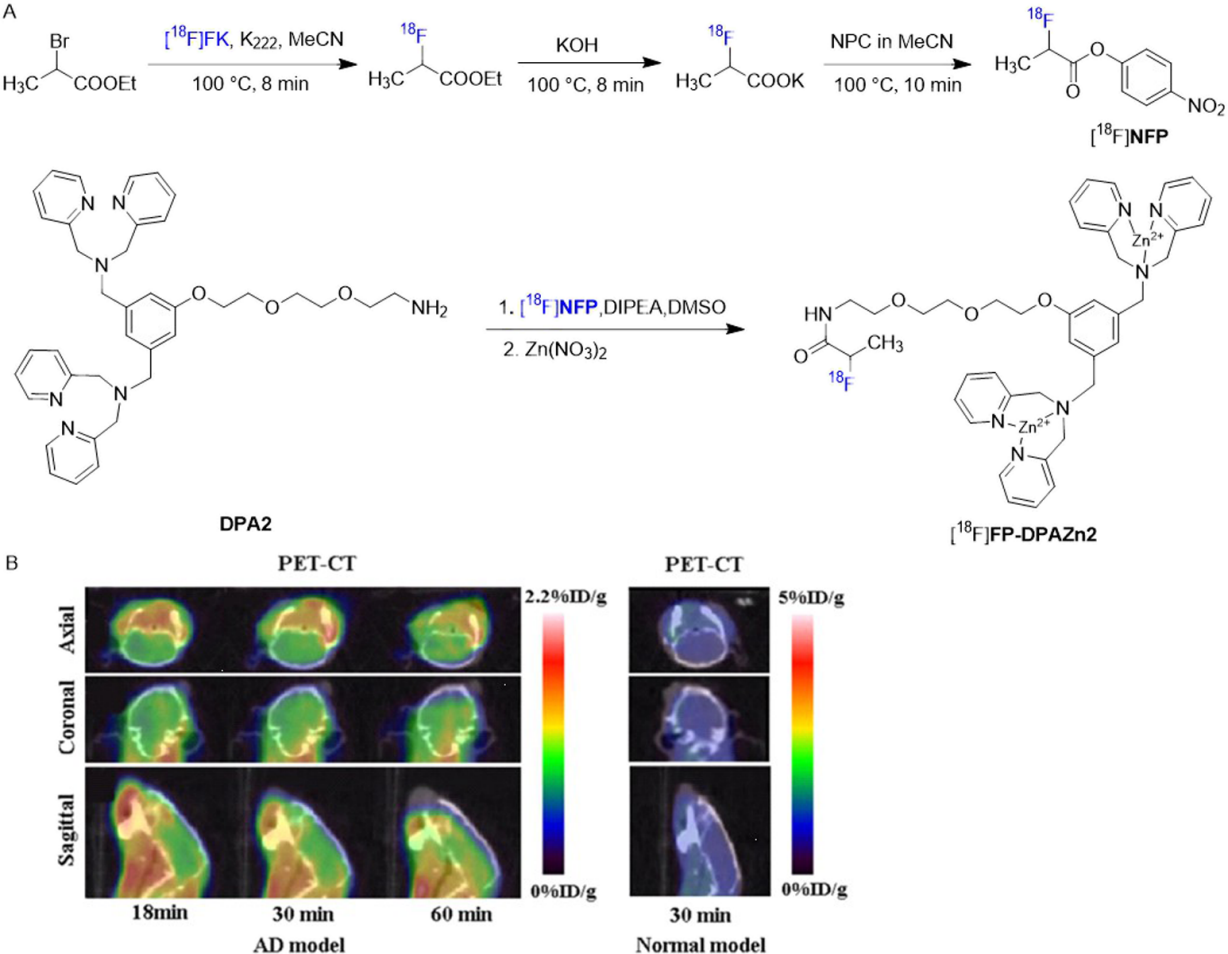
(A) Radiosynthesis of [^18^F]**FP-DPAZn2**, (B) PET-CT images of [^18^F]**FP-DPAZn2** in the brain sections in AD model mice and normal model mice (Reprinted with permission from ref 60, Copyright © 2017 Springer Nature).

## 4 Lessons from Oncology: Cell Death-Targeting PET Tracers with Blood–Brain Barrier Penetration

Although PET radiotracers specifically designed for imaging cell death in neurodegenerative diseases remain limited, significant progress has been made in oncology, where several tracers have been developed to detect various forms of cell death. Importantly, some of these agents demonstrate the ability to cross BBB, making them potential candidates for adaptation or inspiration in the neurodegeneration context. This section highlights selected oncology-derived PET tracers that may inform future strategies for imaging neuronal cell death.

### 4.1 Caspase

Building on their earlier work,^61,62^ Mach and co-workers developed an caspase-3-targeted isatin analog [^18^F]**WC-4–35** which demonstrated 2.20 ± 0.12% ID/g of brain uptake in mice 30 min after tracer injection, though this PET tracer was designed to investigate capase-3 activation in response to cancer treatment.^63^ [^18^F]**WC-4–35** was radiosynthesized in two steps including the radiolabeling of ditosylate precursor and the following reaction with the phenol precursor (Figure 10). Radiochemical purity and the specific activity are 99.9% and 996 mCi/μmol, respectively. [^18^F]**WC-4–35** managed to reflect capase-3 activation both *in vitro* and *in vivo*. However, the tracer exhibited high nonspecific retention, likely due to its strong cell membrane permeability.

**Figure 10:**
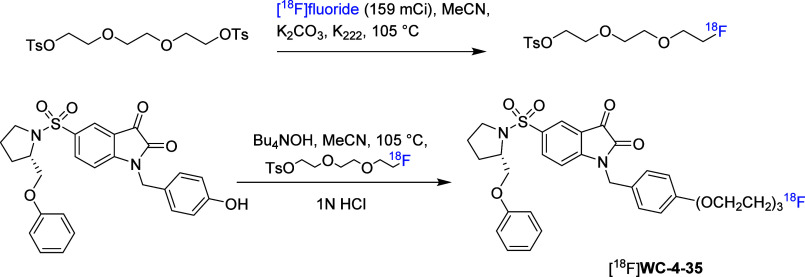
Radiosynthesis of [^18^F]**WC-4–35**.

Lin and colleagues developed a stimuli-responsive PET tracer [^18^F]­DEVD-Cys­(StBu)-PPG­(CBT)-AmBF_3_ ([^18^F]**4**) for imaging drug-induced tumor apoptosis (Figure 11A).^64^ The tracer design incorporates the caspase-cleavable peptide sequence Asp-Glu-Val-Asp (DEVD, blue), a disulfide-protected cysteine (pink), 2-cyanobenzothiazole (CBT, green), and [^18^F]­ammoniomethyl-trifluoroborate ([^18^F]­AMBF_3_, orange) conjugated to a glycine side chain. Inside apoptotic cells, caspase-3/7-dependent cleavage releases the DEVD peptide, while thiol-mediated reduction liberates the protected cysteine (Figure 11B). The exposed 1,2-aminothiol group and the CBT’s 2-cyano group undergo a biorthogonal condensation reaction to form amphiphilic dimers, which self-assemble into nanoparticles through π–π stacking. This process leads to high local accumulation of fluorine-18 radioactivity. Tracer [^18^F]**4** was synthesized in a single step within a hot cell, achieving a RCY of 53 ± 6% (decay-corrected to the end of synthesis), radiochemical purity >99%, and a specific activity of 1.45 ± 0.4 Ci/μmol (n = 13). [^18^F]**4** demonstrated good stability in phosphate-buffered saline (PBS) and mouse serum, likely due to the disulfide bond in its structure. *In vivo* μPET imaging of mice bearing HeLa xenografts demonstrated effective tumor localization after systemic administration (Figure 11C). Moreover, PET imaging indicated that the tracer could cross the BBB, highlighting its potential utility for imaging neurodegenerative diseases. Importantly, PET imaging also demonstrated that [^18^F]**4** is capable of crossing the BBB. This characteristic, coupled with its caspase-3/7 specificity and intracellular self-assembly mechanism, suggests potential applicability in detecting apoptosis associated with neurodegenerative diseases, particularly in regions where BBB integrity is compromised, as often observed in the early stages of AD and FLD.

**Figure 11:**
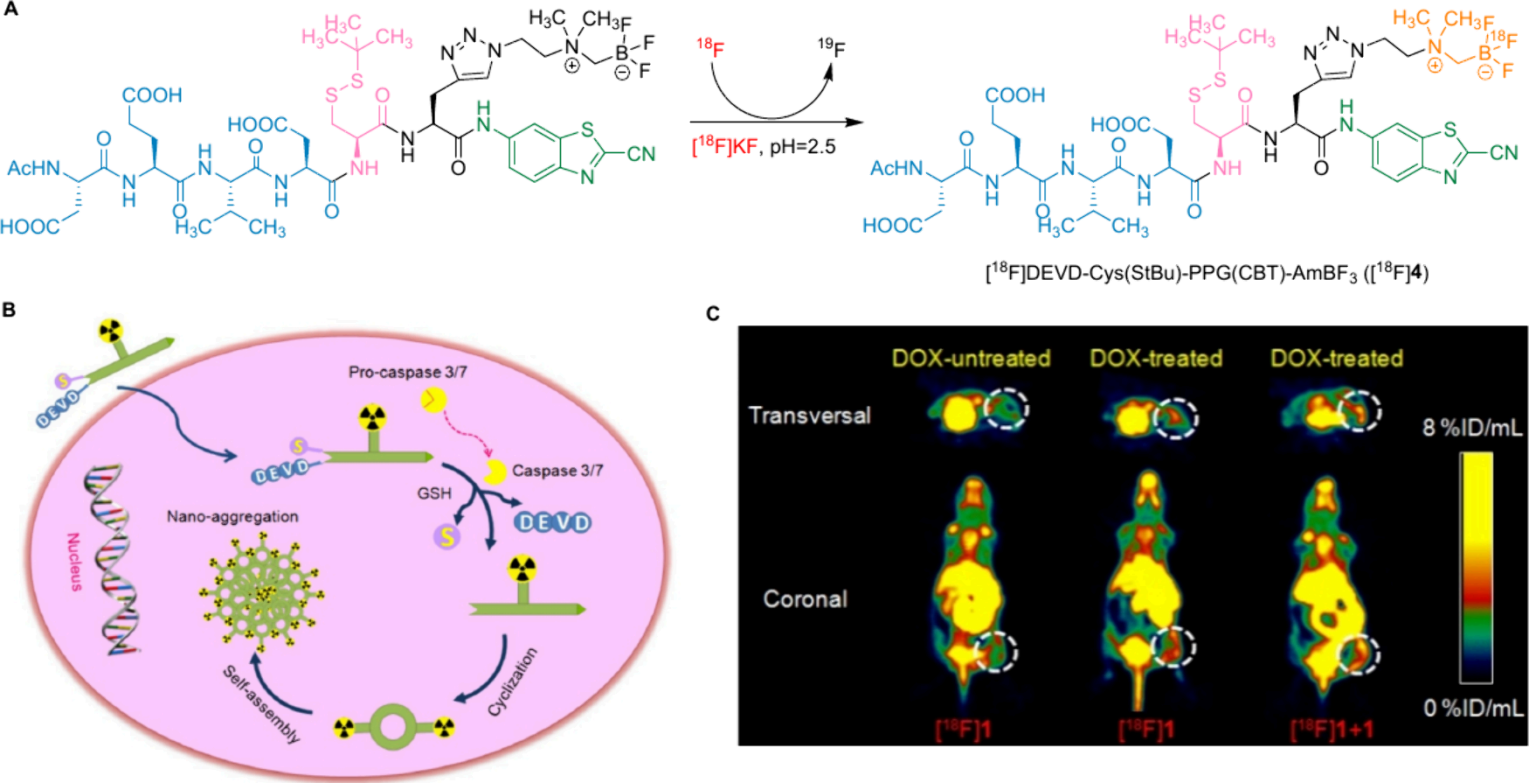
(A) The radiosynthesis of [^18^F]­DEVD-Cys­(StBu)-PPG­(CBT)-AmBF_3_, (B) Proposed mechanism for [^18^F]­DEVD-Cys­(StBu)-PPG­(CBT)-AmBF_3_ targeting apoptotic cells, and (C) Whole-body μPET imaging of [^18^F]­DEVD-Cys­(StBu)-PPG­(CBT)-AmBF_3_ in mice bearing HeLa xenograft tumor (Reprinted with permission under CC BY 4.0 from ref 64, Copyright © 2019 Ivyspring International Publisher).

### 4.2 Poly­(ADP-ribose) Polymerase 1 (PARP1)

Poly­(ADP-ribose) polymerase 1 (PARP1), the most abundant and well-studied member of the PARP family, is an enzyme best known for its critical role in the repair of single-strand DNA breaks through the base excision repair pathway.^65^ Upon sensing DNA damage, PARP1 catalyzes the transfer of ADP-ribose units from NAD^+^ to target proteins, generating long and branched poly­(ADP-ribose) (PAR) chains, which recruit DNA repair machinery to the site of damage. Beyond its canonical function in DNA repair, PARP1 has emerged as a key mediator of a unique form of programmed cell death known as parthanatos.^65–68^ In this pathway, excessive PARP1 activation, often in response to severe oxidative or genotoxic stress, leads to overproduction of PAR polymers at the expense of cellular NAD^+^ and ATP pools. This catastrophic depletion of metabolic stores results in a critical energy deficit, ultimately leading to cell death. Importantly, PAR itself acts as a signaling mediator in parthanatos, translocating to mitochondria and triggering the release of apoptosis-inducing factor (AIF), which then induces large-scale DNA fragmentation and chromatin condensation independent of caspases. Unlike classical apoptosis, parthanatos is characterized by its reliance on PAR polymer formation and is implicated in a range of pathological processes.

PARP1-mediated parthanatos has been increasingly recognized as a contributor to neuronal loss in various neurodegenerative diseases, including AD, PD, HD, and ALS.^69,70^ The involvement of PARP1 in both the DNA repair and parthanatos pathways underscores its dual-edged role in neuronal homeostasis and pathology. While PARP1-targeted PET imaging has primarily been developed for oncology applications to date,^71,72^ its relevance in neurodegenerative diseases, particularly through parthanatos, makes it an appealing target for advancing neuroimaging of cell death.^73^

Cai’s group reported the first BBB-permeable PARP1 PET radioligand [^11^C]**PyBic** (Figure 12A), which was evaluated in both the RG2 rat glioma model and healthy NHPs.^74^ As shown in Figure 12A, [^11^C]**PyBic** was synthesized via radiolabeling of the demethylated precursor with [^11^C]­CH_3_I, achieving decay-corrected radiochemical yields of 43 ± 10% (n = 8). High radiochemical purity (>97%) and good molar activity (148 ± 85 MBq/nmol, n = 6) were also obtained for this tracer. In the RG2 rat glioma model, [^11^C]**PyBic** demonstrated PARP-specific binding within tumor tissue. Importantly, PET imaging in NHPs showed fast and high specific brain uptake in the monkey brain with SUVs ranging from 0.5 to 1.5 (Figure 12B) and displayed high nondisplaceable binding potential (BP_ND_, > 3), underscoring the radioligand’s effective BBB penetration. These promising results suggest that [^11^C]**PyBic** serve as a foundational tool for investigating PARP1-mediated parthanatos in neurodegenerative disease models, warranting further preclinical and translational evaluation.

**Figure 12:**
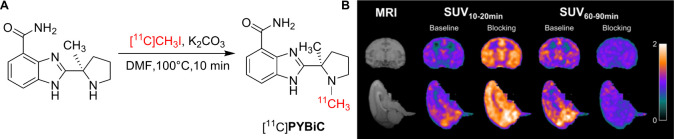
(A) Radiosynthesis of [^11^C]**PyBic**, (B) Brain uptake of [^11^C]**PyBic** in monkey (Reprinted with permission from ref 74, Copyright © 2023 Springer-Verlag GmbH Germany, part of Springer Nature).

While [^11^C]**PyBic** represented the first reported PARP inhibitor–based PET radiotracer with BBB permeability, its development did not progress substantially beyond preclinical evaluation. In contrast, substantial progress was achieved with [^18^F]­FluorThanatrace ([^18^F]**FTT**) (Figure 13), a PARP1-targeting tracer derived from the olaparib scaffold. [^18^F]**FTT** demonstrated effective imaging of PARP1 expression in human cancers,^75,76^ and has advanced to clinical, where it exhibited strong tumor uptake in PARP1-overexpressing malignancies, representing a pivotal milestone in the clinical translation of PARP1-targeted PET imaging.^77,78^ Similarly, the olaparib-derived radiotracer [^18^F]**PARPi** (Figure 13), reported by Reiner et al., exhibited good binding affinity for PARP1 (IC_50_ = 2.8 ± 1.1 nM) and demonstrated selective accumulation in glioblastoma lesions in mouse models.^79,80^ This uptake pattern is likely influenced by both variable PARP1 expression and compromised BBB integrity in the tumor microenvironment, while healthy brain tissue, where the BBB remains intact, showed minimal tracer retention. Notably, [^18^F]**PARPi** has progressed to clinical imaging of human head and neck cancer, where it enabled detection of primary and metastatic lesions with favorable tumor-to-background contrast and without safety concerns.^81^ Together, these olaparib-derived tracers highlight the clinical viability of PARP1-targeted PET imaging, with potential extensions into neurodegenerative contexts, particularly in cases of compromised BBB or upregulated PARP1 expression.

**Figure 13:**
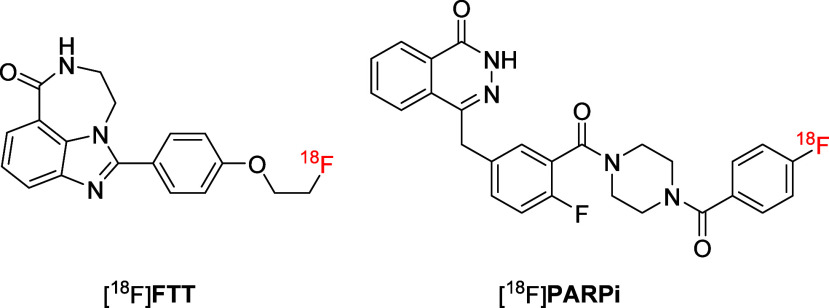
Structures of [^18^F]**FTT** and [^18^F]**PARPi**.

In 2022, Hamerlik and co-workers reported the development of [^11^C]**AZD9574** (Figure 14A), a novel radiolabeled PARP inhibitor Palacaparib (AZD9574).^82,83^ This tracer demonstrated robust BBB penetration and target-specific binding in both preclinical and initial clinical settings, positioning [^11^C]**AZD9574** as a leading candidate for neuro-oncological and neurodegeneration-focused PET imaging. In parallel, our group described the successful preparation of ^18^F-isotopologue of AZD9574 ([^18^F]**AZD9574**, Figure 14A)^84,86^ Cellular uptake studies, autoradiography in rodent and NHP brain tissues, and in vivo PET imaging in NHPs demonstrated that [^18^F]**AZD9574** effectively binds to PARP1 and readily crosses the BBB. It displayed moderate-to-good brain penetration in NHP with the highest radioactivity uptake in the cerebellum (at 1.20 SUV) at 30 min post injection (Figure 14B). An excellent binding specificity was later proved as the radioactivity accumulation in the cerebellum declined by 63.4% upon the high dose (0.5 mg/kg) blocking of AZD9574 (Figure 14B). The analysis of BP_ND_ values also showed the highest uptake in the cerebellum and demonstrated a similar distribution pattern as the expression of PARP1 in the brain. Additionally, significant reduction of BP_ND_ values were observed under blocking conditions. These results highlight the potential of [^18^F]**AZD9574** as a promising tool for visualizing PARP1 in the brain and investigating its role in various neurological diseases. Moreover, an analog named [^11^C]**AZ14193391** was also prepared by Leo and co-workers with 1.5 SUV in brain at *C*_max_.^87^

**Figure 14:**
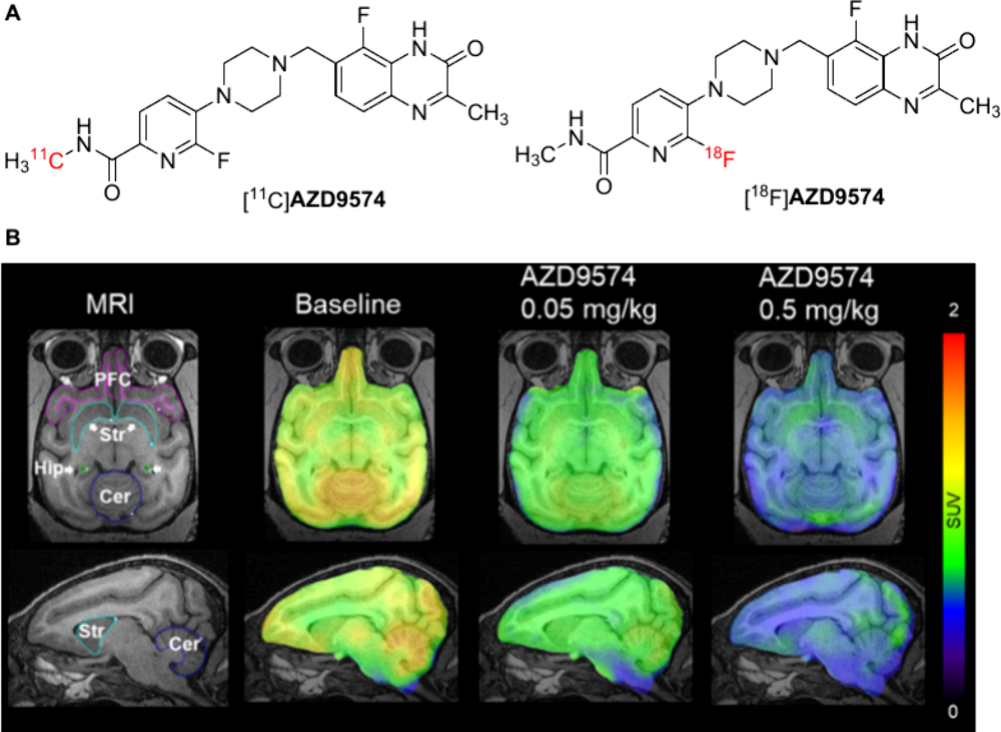
(A) The structures of [^11^C]**AZD9574** and [^18^F]**AZD9574**, (B) PET imaging of [^18^F] **AZD9574** in rhesus monkey brains (Reprinted with permission from ref 86, Copyright © 2025 Elsevier B.V., its licensors, and contributors.).

### 4.3 Phosphatidylethanolamine (PE)

Phosphatidylethanolamine (PE) is another amino phospholipid that undergoes redistribution during various forms of cell stress and death. Unlike PS, PE externalization is more closely associated with late apoptotic or necrotic events and may be upregulated in membrane remodeling processes observed in neuroinflammation. Several studies have demonstrated PE exposure in activated microglia and degenerating axons in multiple sclerosis and traumatic brain injury models, raising the possibility that PE-binding tracers could serve as general indicators of membrane disintegration in chronic neurodegeneration.^88,89^ Duramycin (Figure 14), a 19-amino-acid tetracyclic polypeptide, exhibits high affinity and remarkable specificity for PE, making it a particularly attractive scaffold for the development of PET radiotracers.^89^ Its binding is mediated by a combination of ionic interactions, specifically between the carboxylate group of Asp15 in duramycin and the ammonium group of the PE headgroup, and hydrophobic interactions between the duramycin side chains and the fatty acyl chains of PE.^90–92^ These molecular features confer excellent selectivity and strong binding affinity. Furthermore, duramycin offers additional advantages for tracer development, including its relatively low molecular weight, inherent stability against enzymatic degradation, and ease of radiolabeling with various isotopes.^89^ Several duramycin-based PET tracers, radiolabeled with isotopes such as ^18^F and ^6^ ^8^Ga (Figure 15), have been successfully developed and validated for in vivo imaging of cell death. However, despite these advances, one significant limitation remains: these tracers have demonstrated poor BBB penetration, which restricts their applicability for imaging neuronal cell death in neurodegenerative diseases.^88,93^ Therefore, while PE is conceptually intriguing as a late-stage death marker, current tracers are unlikely to offer meaningful neuroimaging readouts without enhanced delivery platforms or novel binding scaffolds. Future efforts to enhance duramycin-based tracer BBB permeability, potentially via conjugation with carrier peptides or receptor-mediated transcytosis strategies, are critical for enabling neuroimaging applications.

**Figure 15:**
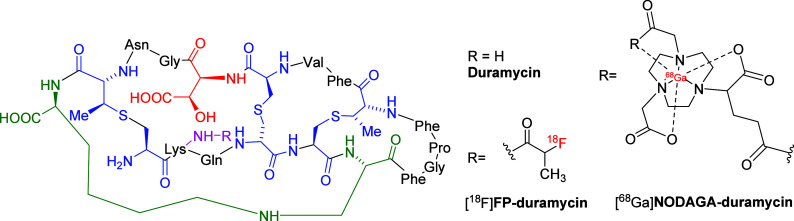
Schematic structures of duramycin and representative duramycin-based PET tracers.

## 5 Conclusion and Perspectives

The development of PET radiotracers for imaging cell death in the brain has advanced significantly over the past decade, with notable progress in tracers targeting key apoptotic and necroptotic markers such as caspases and RIPK1. Isatin sulfonamide derivatives, exemplified by [^18^F]**1–3** and [^18^F]­WC-4–35, have demonstrated promising affinity and selectivity for caspase-3/7, though their application to neurodegenerative disease models remains limited by challenges including BBB penetration and signal specificity. Similarly, RIPK1-targeting radiotracers, such as [^18^F]­CNY-07, [^11^C]­CNY-10, and [^11^C]­TZ7774, have provided encouraging proof-of-concept data, particularly in neuroinflammatory and neurodegenerative models, yet further optimization of binding specificity, pharmacokinetics, and translational robustness is needed. While the majority of tracers described herein were initially developed within oncological contexts, increasing recognition of the central role of cell death pathways in neurodegeneration has catalyzed their adaptation for brain imaging. Emerging dual-modality tracers, such as [^68^Ga]­Ga-TC3-OGDOTA, highlight the potential of multimodal approaches to overcome the inherent limitations of PET alone, enabling complementary spatial and molecular insights.

Importantly, several cell death–targeting PET probes have already entered clinical evaluation, underscoring their translational promise. The apoptosis tracer [^18^F]­ML-10 demonstrated uptake in infarcted brain regions of stroke patients, though its use was limited to areas with BBB disruption. In oncology, PARP-targeting tracers such as [^18^F]­FTT, [^18^F]­PARPi and [^11^C]­PyBic have shown favorable tumor-to-background ratios in oncology applications. More recently, [^18^F]­AZD9574, derived from a clinically validated PARP inhibitor, has exhibited robust brain penetration and target engagement in early human imaging studies, suggesting strong potential for neurological applications. These encouraging results highlight the feasibility of safely translating cell death tracers to patients and provide valuable benchmarks for their future deployment in neurodegenerative disease contexts.

Looking ahead, several key challenges remain. First, the heterogeneous and dynamic nature of neurodegenerative pathology necessitates tracers with high target selectivity, excellent BBB permeability, and favorable kinetic profiles. Second, balancing metabolic stability with rapid clearance of nonspecific signals will be critical for maximizing image contrast and diagnostic accuracy. Finally, as the molecular understanding of neurodegeneration deepens, novel targets are expected to emerge.

In conclusion, although significant strides have been made in PET tracer development for imaging cell death in the brain, there remains ample opportunity to refine existing radiotracers, explore novel molecular targets such as dual leucine zipper kinase (DLK),^94,95^ and expand clinical applications. The convergence of chemistry, molecular biology, and imaging technology is poised to unlock new diagnostic and therapeutic possibilities in neurodegenerative disease. Moreover, as understanding of regulated cell death pathways such as ferroptosis and PANoptosis evolves, exploration of corresponding molecular targets may unveil next-generation neuroimaging strategies.

## ■ Abbreviation

AD: Alzheimer’s disease
ALS: amyotrophic lateral sclerosis
BBB: blood–brain barrier
BPND: nondisplaceable binding potential
CBT: 2-cyanobenzothiazole
CJD: Creutzfeldt–Jakob disease
CNS: central nervous system
DLK: dual leucine zipper kinase
EOB: end of bombardment
FTLD: frontotemporal lobar degeneration
FTT: FluorThanatrace
HD: Huntington’s disease
LBD: Lewy body dementia
MCAO: middle cerebral artery occlusion
MRI: magnetic resonance imaging
NHP: nonhuman primates
PAR: poly­(ADP-ribose)
PARP: poly­(ADP-ribose) polymerase
PBS: phosphate-buffered saline
PD: Parkinson’s diseases
PE: phosphatidylethanolamine
PET: positron emission tomography
PS: phosphatidylserine
RCY: radiochemical yield
RIPK1: receptor-interacting protein kinase 1
ROI: regions of interest
TNFR1: tumor necrosis factor receptor 1
μPET: microPET
WT: wild-type
Zn2+-DPA: Zn2+-2,2’-dipicolylamine
